# Alcohol dependence and driving: knowledge of DVLA regulations

**DOI:** 10.1192/pb.bp.113.045963

**Published:** 2015-02

**Authors:** Andrew Collier, Maggie Watts, Sujoy Ghosh, Peter Rice, Neil Dewhurst

**Affiliations:** 1 Ayr Hospital, Ayr; 2 Department of Public Health, NHS Ayrshire and Arran; 3 Stracathro Hospital, Brechin; 4 Royal College of Physicians, Edinburgh

## Abstract

**Aims and Methods** The UK’s Driver Vehicle Licensing Authority (DVLA) requires individuals to report if they have a medical condition such as alcohol dependence. General Medical Council guidance indicates that medical practitioners should ensure patients are aware of their impairment and requirement to notify the DVLA.

**Results** In a survey of 246 people with known alcohol dependence, none were aware of advice on driving given by medical practitioners and none had self-reported. In addition, 362 doctors, either attending a college symposium or visiting a college website, were asked about their knowledge of DVLA regulations regarding alcohol dependence: 73% of those attending the symposium and 63% of those visiting the website answered incorrectly. In Scotland, over 20 000 people have alcohol dependence (over 1 million people with alcohol abuse), yet only 2548 people with alcohol problems self-reported to the DVLA in 2011.

**Clinical implications** If the DVLA regulations were implemented, it could make an enormous difference to the behaviours of the driving public.

The dangers of driving while under the influence of alcohol are well known. Alcohol remains a significant public health risk and has been identified as the most important factor contributing to the occurrence of severe to fatal automobile crashes.^1^ Acute alcohol intoxication affects the behavioural and coordinating functions necessary for driving.^2^ Alcohol consumption at lower levels also interferes with performance on neurological and psychological tasks,^3^ which include a wide variety of cognitive processes,^4^ affects immediate memory span and short-term memory^5^ as well as motor speed and coordination.^6^

Driver licensing in Great Britain is governed by the third European Commission Directive on the Driving Licence.^7^ Annex III of the directive provides the minimum medical standards for driving expected across all member states. It states: ‘Driving licences shall not be issued to, or renewed for, applicants or drivers who are dependent on alcohol or unable to refrain from drinking and driving. After a proven period of abstinence and subject to authorised medical opinion and regular medical check-ups, driving licences may be issued to, or renewed for, applicant or drivers who have in the past been dependent on alcohol’.^7^ The persistent misuse of drugs or alcohol, whether or not misuse amounts to dependency, is a relevant disability in the Motor Vehicles (Driving Licences) Regulations 1999.

Guidance from the Driver and Vehicle Licensing Agency (DVLA) states that persistent alcohol misuse requires licence revocation or refusal until a minimum 6-month period of controlled drinking or abstinence has been obtained, with normalisation of blood parameters. Alcohol dependency requires licence revocation or refusal until a 1-year period free from alcohol problems.^8^ Abstinence will normally be required and medical reports from the driver’s general practitioner (GP) are necessary, usually in conjunction with an independent medical report.

The aim of this study was to ascertain the knowledge of ‘recovering alcoholics’ of the DVLA regulations related to driving a car while still ‘actively drinking’ and whether they could recollect whether their health professional had given them appropriate advice. In addition, a total of 362 senior doctors, either attending a Royal College of Physicians (Edinburgh) symposium or visiting a Royal College of Physicians (Edinburgh)/Royal College of Psychiatrists (Scotland) website, were asked about their knowledge of DVLA regulations and alcohol dependence. The terms ‘recovering alcoholics’ and ‘actively drinking’ are recognised and regularly used by Alcoholics Anonymous.^9^

## Method

### Patients

In total, 246 ‘recovering alcoholics’ attending five different Alcoholics Anonymous (AA) meetings in Ayrshire, Scotland were surveyed during 2011. The individuals surveyed had all previously been heavy consumers of alcohol (> 100 units/week). They had attended AA meetings regularly for over 6 months and by self-report had been free of any alcoholic intake for at least this period. Membership of the Fellowship of AA is described in Tradition 3: ‘The only requirement for A.A. membership is a desire to stop drinking’.^9^ (Traditions and ‘Steps’ are the foundation of AA.) Of the sample, 194 (79%) were male and the ages ranged from 21 to 80 years, median 57 years. As anonymity is a very important aspect of AA, further demographic detail was not collected. All individuals questioned were in possession of a current driving licence and had the willingness and apparent capacity to provide consent for participation in the survey. They were asked to complete a brief, anonymous questionnaire (unsupervised) about their knowledge of DVLA regulations and about the information that they had been given by health professionals. Those involved in any drink driving offences were excluded. The survey was undertaken with the full knowledge of Fellowship of AA. However, as according to the AA Tradition 6: ‘Our groups ought never endorse, finance or lend our name to any related facility or outside enterprise, lest problems of money, property and prestige divert us from our primary purpose’,^9^ the work was conducted independently of the Fellowship. The questionnaires were distributed to members for completion before or immediately after AA meetings (Table 1).

**Table 1:** Participant survey results

Question	Yes, *n* (%)
1 Are you a recovering alcoholic?	246 (100)
	
2 Do you hava a driving licence?	246 (100)
	
3 Do you drive a car?	246 (100)
	
4 Did you problems with alcohol lead you to see your GP?	185 (71)
	
5 Do you recollect your GP giving you any advice about driving?	0
	
6 Have you ever been admitted to hospital due to your alcohol problems?	137 (56)
	
7 When you were in hospital do you recollect getting any advice about driving?	0
	
8 Do you recollect if your GP or a hospital doctor have ever asked you/told you to stop driving?	0
	
9 Have you ever informed the DVLA that you have/had a problem with alcohol?	0
	
10 Do you think that it would be sensible/reasonable to be told not to drive if you still had an active problem with drinking?	223 (91)
	
11 Have you ever driven a car knowing that you were under the influence of alcohol/were over the limit (including the morning after)?	246 (100)
	
12 Have you ever driven a car when you were withdrawing from alcohol?	194 (79)
	
13 Did you ever stop driving or think of not driving while still actively drinking?	103 (42)

GP, general practitioner.

### Doctors

There were nine questions posed to doctors at the symposium and online: three were based on alcohol-related problems, three on cardiology, two on psychiatric issues and one on diabetes. Of the physicians who attended the Royal College of Physicians (Edinburgh) symposium, 292 took part in the survey and 70 doctors did so online.

The alcohol and driving question stated: ‘If a patient is admitted to hospital with an alcohol dependence problem (requiring benzodiazepine treatment for withdrawal symptoms) the patient must be advised to inform the DVLA, who will revoke their licence for a year’. Respondents could answer that this was true, false or that they did not know the answer.

## Results

The 246 ‘recovering alcoholics’ answered all 13 questions (Table 1). All 246 had a driving licence and drove a car; 185 (71%) went to see their GP with an alcohol problem and 137 (56%) were admitted to hospital with alcohol problems. None recollected their GP or a hospital doctor asking them to stop driving. In addition, none had informed the DVLA that they had a problem with alcohol.

At the symposium, 73% physicians answered the question incorrectly and on the website 62% of physicians and psychiatrists gave an incorrect answer; 9% of those at the symposium indicated that they did not know the answer. With regard to questions on alcohol-related seizures and alcohol misuse, 64% and 62% respectively answered incorrectly. The other non-alcohol related questions demonstrated a higher level of knowledge among the symposium audience (13–42% incorrect).

## Discussion

This study has shown that awareness of individual responsibility among recovering alcohol-dependent individuals is low and recollection of discussion with health professionals about driving is non-existent. The people we surveyed were regular attendees of AA meetings. This is likely to be a group who have developed a high level of understanding and insight into their alcohol problem and would be willing to engage in open discussion with doctors. A few specialist alcohol services in Scotland provide written information to newly referred patients alerting them to their responsibilities regarding the DVLA, but it is not known whether patients recall this (in the short or long term) and clearly very few act on the advice. It is also probable that a significant proportion of health professionals are not aware of the advice that they should be giving in relation to driving and alcohol dependency and alcohol misuse. These small but significant doctor surveys reflect that knowledge of alcohol-related DVLA issues is poor among hospital doctors. The Royal College of General Practitioners (Scotland) was invited to participate but declined this opportunity. General practitioners form a key professional group regarding health issues and driving. This lack of knowledge in ‘recovering alcoholics’ may reflect a number of elements, including lack of knowledge in health professionals, lack of willingness of health professionals to discuss the issue or lack of recollection from the individuals themselves. In addition, it is possible that health professionals who are aware of the appropriate advice are unwilling to discuss the topic in case it would adversely affect their relationship with the patient.^10,11^ In particular, there may be a reluctance to raise the issue of driving in case this becomes a disincentive for patients to be open about their drinking.

The DVLA guidelines^8^ give advice on a number of medical conditions that may affect driving, including diabetes mellitus, epilepsy, sleep apnoea, dementia, psychiatric disorders, as well as alcohol misuse and alcohol dependency. If a patient is admitted with a ‘first’ seizure, most doctors and health professionals would know to advise the patient that they may not drive for a year.^12^ In addition, if a patient is started on insulin therapy, then most doctors and health professionals, particularly those associated with the management of diabetes,^13^ would know that they should advise their patient to inform the DVLA.

The definition of alcohol misuse used by the DVLA – ‘A state which, because of consumption of alcohol, causes disturbance of behaviour, related disease or other consequences, likely to cause the patient, his/her family or society harm now, or in the future, and which may or may not be associated with dependency’^8^ – is helpful but can be difficult to interpret in the context of dealing with patients in an acute medical setting. The definition of alcohol dependence used by the DVLA – ‘A cluster of behavioural, cognitive and physiological phenomena that develop after

**Table 2:** Notifications to DVLA on UK licence holders with alcohol misuse or alcohol dependence from all sources, 2006–2011^19^

	2006	2007	2008	2009	2010	2011
Self-reported	378	1268	1265	1176	1974	2548
						
Other (e.g. reported by doctor, family member)	41	26	52	24	83	168

DVLA, Driver & Vehicle Licensing Agency.

repeated alcohol use and which include a strong desire to take alcohol, difficulties in controlling its use, persistence in its use despite harmful consequences, with evidence of increased tolerance and sometimes a physical withdrawal state’^8^ – is much clearer and, particularly in relation to alcohol withdrawal, much easier to implement clinically. If more hospital doctors were aware of the DVLA repercussions of making the diagnosis of alcohol dependence or misuse, it may make the use of alcohol withdrawal regimens more problematic. For example, the Clinical Institute Withdrawal Assessment – Alcohol (CIWA–A) scale^14^ is commonly used in alcohol withdrawal situations in medical wards. Perfunctory attention to the history and frequent overreliance on unsubstantiated alcohol use can lead doctors to the inappropriate use of CIWA-A, which could have significant consequences on lifestyle and driving advice they give their patients.^15^

The responsibility for ensuring that the patient informs the DVLA lies with the licence holder. The guidelines for fitness to drive are available on the DVLA website (www.dvla.gov.uk) and are revised every few months. The website states that the General Medical Council (GMC) has issued clear guidelines to doctors with regard to their responsibility to the DVLA:^16^ ‘the DVLA is legally responsible for deciding if a person is medically unfit to drive. They need to know when the driving licence holders have a condition, which may, now or in the future, affect their safety as a driver’. It also states that if a patient has such a condition, the doctor should ‘make sure that the patients understand that the condition may impair their ability to drive’ and ‘explain to patients that they have a legal duty to inform the DVLA about their condition’. In addition, it states that doctors can ‘suggest that the patient seek a second medical opinion, and make the appropriate arrangements for the patient to do so’ if the patient is unwilling to accept the diagnosis (personal communication, DVLA freedom of information request responses 2010/2011). Patients who fail to notify the DVLA of a medical condition without reasonable excuse are guilty of an offence. Failure to notify the DVLA also invalidates their motor insurance cover. Current notification levels of alcohol misuse and alcohol dependency received by the DVLA are very low (Table 2). A small number are also reported through a third party (which may be family, friend, health professional, etc.).

### Situation in Scotland

In the Scottish population, the estimated prevalence of alcohol dependence at 4.9% and of harmful and hazardous use at 27.9% indicates around 20 000 people with dependence and over a million with harmful or hazardous use.^17^ A high proportion of these are likely to be driving licence holders since approximately 86% of the UK adult population has a driving licence of some sort, with 73% being full UK licences.^18^ Most licence holders obtain their licence in young adulthood, before alcohol dependence develops. Therefore it is not unreasonable to estimate that there may be in excess of 150 000 licence holders with alcohol dependence in Scotland alone and a further 700 000 licence holders with harmful or hazardous use of alcohol. This represents an enormous cohort of people who should be reporting their alcohol problems to the DVLA. The Department for Transport report on the attitudes of health professionals giving advice on fitness to drive (including for people with alcohol and drug misuse and dependency) contains a series of recommendations for improving such advice.^19^ Key among these are recommendations on the inclusion of a question on fitness to drive in the exit examination for all relevant medical specialties, the creation of clear, well-signposted guidelines for use in general practice and the production of a clear flowchart for common medical conditions to which healthcare practitioners can refer.

### Limitations

There were some limitations to the study. The questionnaires had not been externally validated but the questions were simple, straightforward and easy to understand. We accept that the group of ‘recovering alcoholics’ questioned involved only a limited number of adults in Ayrshire. AA meetings provided a venue where motivated individuals who had recognised their problem with alcohol were present; it might be considered that their recall of advice would be greater than that of those who chose not to attend AA. Alternative methods for assessing recall of medical advice, such as surveying patients at addiction clinics, could have suffered from bias as the clinic staff became aware of the study. A presumption was made that none of the attendees experienced alcohol-related brain damage or other memory loss syndrome and could recall accurately the discussions that they had had with their medical advisers about their drinking. The study demonstrated non-existent knowledge of the DVLA regulations relating to alcohol dependence and misuse among a group of ‘recovering alcoholics’ plus a poor level of knowledge in a group of senior doctors. Very importantly, the figures obtained from the DVLA indicate very low levels of self-reporting. During the 2011/2012 period there were 38 737 alcohol-related hospital discharges in Scotland^20^ and almost 97 830 alcohol brief interventions completed,^21^ which give numerous opportunities for alcohol advice. If the DVLA regulations were implemented, it could make an enormous difference to the understanding and behaviours of the driving public.

## References

[1] Calhoun VD Pekar JJ Pearlson GD Alcohol intoxication effects on simulated driving: exploring alcohol-dose effects on brain activation using functional MRI. Neuropsychopharmacology 2004; 29: 2097–17. 15316570 10.1038/sj.npp.1300543

[2] Mitchell MC Alcohol-induced impairment of central nervous system function: behavioral skills involved in driving. J Stud Alcohol Suppl 1985; 10: 109–16. 3862850 10.15288/jsas.1985.s10.109

[3] Parker ES Alkana RL Birnbaum IM Hartley JT Noble EP Alcohol and the disruption of cognitive processes. Arch Gen Psychiatry 1974; 31: 8248. 10.1001/archpsyc.1974.01760180064008 4441250

[4] Mongrain S Standing L Impairment of cognition, risk-taking, and self-perception by alcohol. Percept Mot Skills 1989; 69: 199–210. 2780180 10.2466/pms.1989.69.1.199

[5] Jones BM Memory impairment on the ascending and descending limbs of the blood alcohol curve. J Abnorm Psychol 1973; 82: 24–32. 4730653 10.1037/h0034872

[6] Tarter RE Jones BM Motor impairment in chronic alcoholics. Nerv Syst Dis 1971; 32: 632–6. 5112140

[7] Directive 2006/126/EC of the European Parliament and of the Council. Official J Eur Union 30.12.2006.

[8] Driver and Vehicle Licensing Agency. At a glance guide to the current medical standards of fitness to drive. DVLA, 2009 (http://www.gov.uk/government/publications/at-a-glance).

[9] Alcoholics Anonymous. The Story of How Many Thousands of Men and Women Have Recovered from Alcoholism: ‘The Big Book’ (4th edn). Alcoholics Anonymous, 2003.

[10] Thompson P Nelson D DVLA regulations concerning driving and psychiatric disorders: knowledge and attitudes of psychiatrists. Psychiatr Bull 1996; 20: 323–5.

[11] Wise MEJ Watson JP Postal survey of psychiatrists’ knowledge and attitudes towards driving and mental illness. Psychiatr Bull 2001; 25: 345–9.

[12] Frampton A Who can drive home from the emergency department? A questionnaire based study of emergency physicians’ knowledge of DVLA guidelines. Emerg Med J 2003; 20: 526–30. 14623839 10.1136/emj.20.6.526 PMC1726221

[13] Frier BM Steel JM Matthews DM Duncan LJP Driving and insulin-dependent diabetes. Lancet 1980; i: 1232–4. 6104046 10.1016/s0140-6736(80)91690-6

[14] Shaw JM Kolesar GS Sellers EM Kaplan HL Sandor P Development of optimal treatment tactics for alcohol withdrawal.1. Assessment and effectiveness of supportive care. J Clin Psychopharmacol 1981; 1: 382–9. 7334148 10.1097/00004714-198111000-00006

[15] Hecksel K Bostwick JM Jaeger TM Cha SS Inappropriate use of symptom-triggered therapy for alcohol withdrawal in the general hospital. Mayo Clin Proc 2008; 83: 274–9. 18315992 10.4065/83.3.274

[16] General Medical Council. Confidentiality: Reporting Concerns About Patients to the DVLA. GMC, 2009.

[17] Drummond C Deluca P Oyefeso A Rome A Scrafton S Rice P Scottish Alcohol Needs Assessment. Institute of Psychiatry, King’s College London, 2009.

[18] Driver and Vehicle Licensing Agency (http://www.dft.gov.uk/dvla/pressoffice/stats.asp). Accessed October 2012.

[19] Department for Transport. *The Attitudes of Health Professionals to Giving Advice on Fitness to Drive* (Road Safety Research Report No. 91). Department for Transport, 2010.

[20] ISD Scotland. Alcohol-Related Hospital Statistics Scotland 2011/12 (http://www.isdscotland.org/Publications). Accessed 11th June 2013.

[21] The Scottish Government. Scotland Performs: NHS Scotland (H4: Alcohol Brief Interventions). The Scottish Government (http://www. scotland.gov.uk/About/Performance/scotPerforms/partnerstories/NHSScotlandperformance/alcoholbriefinterventions). Accessed 13th June 2013.

